# “Immune B Cells know it better”: tumorimmunological panel assay to define tumor-associated antigen binding antibodies in patients with metastatic melanomas

**DOI:** 10.1186/1479-5876-13-S1-P16

**Published:** 2015-01-15

**Authors:** Beatrix Kotlan, Timea Balatoni, Katalin Csirbesz, Judit Olasz, Orsolya Csuka, Laszlo Toth, Emil Farkas, Akos Savolt, Andras Szollar, Mihaly Ujhelyi, Szabolcs Horvath, Klara Eles, Miklos Kasler, Francesco M Marincola, Gabriella Liszkay

**Affiliations:** 1 Molecular Immunology and Toxicology, National Institute of Oncology, Budapest, Hungary; 2 Center of Surgical and Molecular Tumorpathology, National Institute of Oncology, Budapest, Hungary; 3 Oncodermatology, National Instute of Oncology, Budapest, Hungary; 4 Pathogenetics, National Instute of Oncology, Budapest, Hungary; 5 Oncosurgery, National Instute of Oncology, Budapest, Hungary; 6 Board of Directors, National Institute of Oncology, Budapest, Hungary; 7 SIDRA Medical and Research Centre, Doha, Qatar

## Background

Revealing novel cancer targeting biomarkers is a great challenge, and especially urging in cancer types with a more pronounced metastatic feature. We focus on potential anti-tumor immune reactions of the host. In order to harness the natural humoral immune response a novel immunological and molecular genetic panel assay has been developed for the investigation of patients with melanomas.

## Materials and methods

Punch biopsies were taken of surgically removed fresh cancerous tissues and peripheral blood was gathered from the patients involved into the study (n= 125). Ethical permission was provided by the Scientific and Research Ethics Committee of the Medical Research Council of the Hungarian Ministry of Health (ETT TUKEB 16462- 02/2010). We established and standardized two experimental strategies (Epstein Barr virus transformation and cloning with limiting dilution assay (LDA) and tumor infiltrating B cell (TIL-B) antibody phage display technology) and started basic processes for a detailed immunglobulin repertoire analysis at DNA level. We set up a novel native tumor cell membrane preparation technique extremely useful for specific detection of tumor reacting antibodies or antibody fragments (eg: immunoblotting, scFv phagemid ELISA). Defining essential tumor-associated antigens on the cancerous tissue specimen by immunohistochemistry became the other part of the tumorimmunological panel assay. Results: We claim that this complex quantitative and qualitative analysis of antibodies in sera and in the tumor microenvironment results in revealing tumorspecific antibodies of human origin. Our antibody profile analysis revealed glycoprotein and sialylated glycolipid based tumor-associated antigen-specific antibody-variable regions in various patterns.

## Conclusions

The present technological developments enable the specific detection of cancer associated sialylated glycolipid and glycoprotein antigens with unique characteristics. The study helps to understand the question of “abnormal glycosilation” and its role in cancer. We conclude that the complex tumorimmunological assay has important potentials in evaluating the host’s anti tumor immune status.

